# Protein Disulfide Isomerase A6 (PDIA6) Restrains Heat-Induced Oxidative Damage in Haemocytes of the Pacific Oyster (*Crassostrea gigas*)

**DOI:** 10.3390/antiox15080963

**Published:** 2026-08-01

**Authors:** Pengcheng Sun, Ming Li, Peng Li, Yang Ma, Lei Gao, Xueshu Zhang, Lingling Wang, Linsheng Song

**Affiliations:** 1 Liaoning Key Laboratory of Marine Animal Immunology & Disease Control, Dalian Ocean University, Dalian 116023, China; pengchengsun123@outlook.com (P.S.); pengli020806@outlook.com (P.L.); mayang122075@outlook.com (Y.M.); gaolei@dlou.edu.cn (L.G.); lshsong@dlou.edu.cn (L.S.); 2 Dalian Key Laboratory of Aquatic Animal Disease Prevention and Control, Dalian Ocean University, Dalian 116023, China; 3 Southern Marine Science and Engineering Guangdong Laboratory (Zhuhai), Zhuhai 519000, China; 4 College of Fisheries, Ocean University of China, Qingdao 266003, China; bioliming@163.com; 5 Zoneco Group Co., Ltd., Dalian 116001, China; 6 Liaoning Key Laboratory of Marine Animal Immunology, Dalian Ocean University, Dalian 116023, China

**Keywords:** PDIA6, *Crassostrea gigas*, heat stress, oxidative stress, apoptosis

## Abstract

Heat stress causes severe oxidative damage and immune cell death in marine bivalves, but its upstream regulators remain unclear. This study identified regulators linking heat stress to oxidative damage in Pacific oyster haemocytes. Under 30 °C exposure, the apoptosis rate of oyster haemocytes increased from ~4.54% to 17.40% at 24 h, accompanied by elevated ROS, malondialdehyde, and lipid hydroperoxide and reduced SOD activity. GSEA and protein interaction analysis of the haemocyte transcriptome pinpointed protein disulfide isomerase A6 (*Cg*PDIA6) as the hub gene linking endoplasmic reticulum stress, apoptosis, and oxidative stress. Single-cell in silico knockout placed *CgPDIA6* at the head of a coupled SOD–peroxiredoxin relay (*Cg*SOD1, *Cg*SOD2, *Cg*PRDX6) and shifted haemocytes toward a stress-activated state. This prediction was confirmed by RNAi knockdown, in which silencing *CgPDIA6* aggravated heat-induced oxidative injury, reduced the expression of antioxidant-related genes (*CgSOD1*, *CgSOD2*, and *CgPRDX6*), and further suppressed SOD activity. Molecular dynamics simulations showed that heat destabilized its catalytic thioredoxin domains, compromising its protective function. These results demonstrate that *Cg*PDIA6 protects haemocytes against heat-induced oxidative damage by sustaining antioxidant enzyme activity, providing insights into redox regulation and heat adaptation in mollusks.

## 1. Introduction

Global warming is driving a sustained rise in sea surface temperatures and a marked increase in the frequency, duration, and intensity of marine heatwaves [1]. For sessile bivalve mollusks, which cannot behaviorally escape heat extremes, elevated temperature disrupts proteostasis, triggers oxidative damage, and compromises immune competence [2]. These effects contribute to mass summer mortality events, causing severe economic losses to the aquaculture industry worldwide [3]. Among the downstream consequences of heat injury, oxidative stress occupies a central pathological role [2]. Heat exposure stimulates mitochondrial electron transport chain dysfunction and uncoupled respiration, which leads to a burst of ROS production [4]. This burst subsequently overwhelms endogenous antioxidant defenses [5]. The resulting redox imbalance drives lipid peroxidation, protein carbonylation, and DNA strand breaks, ultimately committing stressed cells to apoptotic pathways [6]. In bivalves, heat-induced ROS accumulation has been directly associated with haemocyte apoptosis, suppression of superoxide dismutase (SOD) activity, and elevation of malondialdehyde (MDA) and lipid hydroperoxide (LPO) as markers of oxidative injury [7]. Despite the centrality of oxidative stress in bivalve heat pathology, the upstream molecular regulators that couple heat perception to redox dysregulation remain poorly defined.

Protein disulfide isomerase A6 (PDIA6) is an endoplasmic reticulum-resident oxidoreductase that catalyzes disulfide bond formation [8]. It also controls ER redox homeostasis by managing the oxidative folding environment of the ER lumen [9]. PDIA6 operates at the intersection of the unfolded protein response (UPR) and oxidative stress. Under ER stress, PDIA6 binds directly to IRE1α at an oxidized cysteine residue (Cys148) and modulates PERK signaling. This attenuates prolonged UPR activation and limits apoptosis [10]. Loss of PDIA6 has the opposite effect. It causes sustained IRE1α autophosphorylation, excessive *XBP1* splicing, and intracellular ROS accumulation [11]. In contrast, PDIA6 expression stabilizes ER redox balance and suppresses oxidative damage [8]. PDIA6 deficiency also lowers SOD activity and promotes lipid peroxidation (elevated MDA and LPO), while its overexpression reduces H_2_O_2_-induced oxidative stress and cellular senescence [12]. Heat stress accelerates ER protein misfolding and generates aberrant disulfide bonds, placing PDIA6 at the convergence of heat injury pathways [13]. *PDIA6* is strongly upregulated under heat exposure in Pacific oyster *Crassostrea gigas*, sea cucumber (*Apostichopus japonicus*), razor clam (*Sinonovacula constricta*), and Chinese honeybee (*Apis cerana cerana*), suggesting a conserved role in coordinating antioxidant and proteostatic responses to heat stress [14,15,16]. However, whether PDIA6 induction in bivalve immune cells directly reshapes the cellular antioxidant landscape and redox-dependent apoptosis remains experimentally untested.

The Pacific oyster (*C. gigas*) is the most widely farmed bivalve worldwide and a well-established model for studying environmental stress adaptation [17]. With global warming intensifying marine heatwaves, heat-induced mass mortality has become a major constraint on oyster aquaculture, in which haemocytes, the primary immune cells, are especially vulnerable to oxidative damage [18]. Clarifying how oyster haemocytes counter heat-induced oxidative stress is therefore essential to address this challenge. This study investigates how PDIA6 coordinates antioxidant defence in oyster haemocytes under heat stress. The findings provide new insights into redox regulation in marine invertebrates and a candidate target for heat-resilience breeding.

## 2. Materials and Methods

### 2.1. Experimental Animals and Haemocyte Collection

Adult *C. gigas* (shell length 8 ± 2 cm) were collected from a certified farm in Dalian, Liaoning Province, China, and acclimated in aerated, filtered seawater at 16 ± 1 °C for one week [17]. A total of 200 oysters were used in this study. Each biological replicate represented an individual oyster, and samples were collected independently without pooling. All procedures were approved by the Ethics Committee of Dalian Ocean University (Approval Code: DLOU2024030). For heat stress, oysters were subjected to 30 ± 1 °C. Haemocytes were withdrawn from the adductor muscle sinus, mixed 1:1 (*v*/*v*) with anticoagulant buffer, centrifuged at 600× *g* for 5 min at 4 °C using a centrifuge (Eppendorf, Hamburg, Germany), washed three times, and resuspended in modified L-15 medium (Gibco, Grand Island, NY, USA) [19].

### 2.2. Apoptosis Detection and Oxidative Stress Profiling Under Heat Stress

Oysters were subjected to heat stress at 30 °C, a temperature previously shown to induce significant heat stress responses in *C. gigas* [20]. Haemocytes were then collected at 0, 6, 12, 24, and 48 h post-exposure (n = 3 independent oysters per time point), following the procedure described in Section 2.1. Apoptosis was quantified with an Annexin V-FITC/PI kit (Elabscience, Wuhan, China, E-CK-A252) on a BD FACSAria II flow cytometer (≥10,000 events per sample). For oxidative stress profiling, haemocytes from control (16 °C) and heat-stressed (30 °C, 24 h) oysters were assayed for ROS, SOD activity, MDA, and LPO using commercial kits (Shanghai Enzyme-Linked Biotechnology Co., Ltd., Shanghai, China; n = 3 independent oysters per group).

### 2.3. Transcriptome Sequencing and Differential Expression Analysis

Haemocytes from control (16 °C) and heat-stressed (30 °C, 24 h) oysters were collected for transcriptome profiling (n = 3 independent oysters per group). Total RNA was extracted with TRIzol (Thermo Fisher Scientific, Waltham, MA, USA) and integrity was verified on an Agilent 2100 Bioanalyzer (Agilent Technologies, Santa Clara, CA, USA; RIN ≥ 7.0). Paired-end libraries (150 bp) were sequenced on an Illumina NovaSeq 6000 (Illumina, Inc., San Diego, CA, USA). Clean reads (fastp v0.23.0) [21] were aligned to the *C. gigas* genome (xbMagGiga1.1, NCBI) with HISAT2 (v2.0.5) [22], and expression was quantified as FPKM with StringTie (v2.1.4). DEGs were identified with DESeq2 (v1.46.0) in R (v4.5.1; |log_2_FC| ≥ 1, FDR < 0.05) and visualized as volcano plots (ggplot2).

### 2.4. Functional Enrichment and Pathway Analysis

KEGG enrichment of DEGs was performed with clusterProfiler in R v4.5.1 (FDR < 0.05) [23]. GSEA was run on all expressed genes ranked by log_2_FC against KEGG gene sets, retaining pathways with FDR < 0.05 and |NES| > 1.0. The top 10 enriched pathways are shown as bubble plots.

### 2.5. Protein–Protein Interaction Network Construction and Hub Gene Identification

Candidate genes were defined as the union of the top 100 high-confidence DEGs (STRING v12.0, score ≥ 0.4) [24], leading-edge genes from the top 20 GSEA pathways, and all genes from the top 10 KEGG pathways. The PPI network was retrieved from STRING and visualized in Cytoscape (v3.10.0) [25]. Hub genes were ranked by node degree, betweenness centrality, and co-expression correlation. PDIA6 ranked highest, based on its central connectivity across the ER stress, protein processing, and oxidative stress subnetworks and its interactions with effectors such as ERO1B and GPX2-like.

### 2.6. Sequence Analysis, Domain Architecture, Structural Modeling, and Phylogenetic Analysis of CgPDIA6

The mRNA sequence and relevant structural information of *CgPDIA6* were retrieved from NCBI, and the schematic diagram of mRNA architecture was generated using DOG 2.0 (Domain Graphics) [26]. Conserved functional domains of the deduced *Cg*PDIA6 protein were predicted via the SMART database (http://smart.embl-heidelberg.de/) and visualized with DOG 2.0. The amino acid sequence of *Cg*PDIA6 was downloaded from NCBI, and its three-dimensional protein structure was predicted on the AlphaFold3 server (https://alphafoldserver.com/) [27]. The predicted structural model was further rendered and refined in PyMOL (https://www.pymol.org/, Schrödinger, LLC, New York, NY, USA). For phylogenetic analysis, PDIA6 protein sequences from 27 representative species were collected from NCBI. A phylogenetic tree was constructed in MEGA 11 using the neighbor-joining algorithm with 1000 bootstrap replicates for reliability evaluation [28].

### 2.7. Tissue-Specific and Developmental Expression Profiling

FPKM data for *CgPDIA6* and antioxidant-related genes were obtained from a public *C. gigas* RNA-seq dataset (PRJNA146329). Tissue-specific expression was visualized as heatmaps (pheatmap, R), and developmental dynamics of *CgPDIA6* as radar charts (ggradar, R).

### 2.8. In Silico PDIA6 Knockout and Single-Cell Population Analysis

Single-cell RNA-seq data of *C. gigas* haemocytes (ERX12134208) were clustered with Seurat (v4.3.0) and trajectory-analyzed with Monocle3 (v1.3.1), yielding 13 clusters (0–12) on UMAP [29]. In silico knockout was simulated by computationally removing all PDIA6-associated interaction edges, and cluster composition and marker expression were compared between wild-type and knockout configurations. KEGG enrichment of the top 5000 highly variable genes under knockout was performed with clusterProfiler [23]. A secondary PPI network was built from the highly expressed genes of the most-shifted clusters to resolve downstream antioxidant and stress-response dependencies regulated by *Cg*PDIA6.

### 2.9. RNA Interference-Mediated Knockdown of CgPDIA6

For RNA interference experiments, dsRNA expression vectors targeting *CgPDIA6* were constructed and introduced into RNase III-deficient *Escherichia coli* HT115 (DE3). The dsRNA fragments of *CgPDIA6* (dsPDIA6) and EGFP (dsEGFP, negative control) were induced by IPTG. Each oyster received an injection of 10 μg dsRNA in 100 μL sterile seawater into the adductor muscle sinus, with heat stress at 30 °C initiated simultaneously with dsRNA injection. Knockdown efficiency was verified by qRT-PCR at 0, 6, 12, and 24 h post-injection under both 16 °C (baseline) and 30 °C (heat stress) conditions [30].

### 2.10. Quantitative Real-Time PCR (qRT-PCR)

Total RNA was extracted from haemocytes with TRIzol and reverse-transcribed using the TransScript One-Step gDNA Removal and cDNA Synthesis SuperMix (TransGen Biotech, Beijing, China). Quantitative PCR was performed with NovoStart SYBR qPCR SuperMix Plus (Novoprotein, Shanghai, China) on a CFX96 system (Bio-Rad, Hercules, CA, USA), using CgEF as the internal reference and gene-specific primers (Supplementary Table S1). Relative expression was calculated by the 2^−ΔΔCT^ method with three biological and three technical replicates per group.

### 2.11. Oxidative Stress Parameter Assays Following RNAi Knockdown

To evaluate the functional role of *CgPDIA6* in restraining heat-induced oxidative damage, haemocytes were collected from two experimental groups at 0, 6, 12, and 24 h under 30 °C heat stress: (1) dsEGFP-injected controls and (2) dsPDIA6-injected knockdown oysters. Intracellular ROS activity, SOD activity, LPO content, and MDA content were quantified using commercial detection kits (Shanghai Enzyme-Linked Biotechnology Co., Ltd., Shanghai, China) following the manufacturers’ protocols. All assays were performed with three biological replicates per group.

### 2.12. Molecular Dynamics Simulation

Molecular dynamics simulations of *CgPDIA6* were performed using GROMACS (v2025.4) with the AMBER99SB-ILDN force field and the TIP3P water model [31]. The protein was solvated in a dodecahedral box with a 1.5 nm margin and neutralized with 0.15 M NaCl. After energy minimization and NVT/NPT equilibration, 100 ns production simulations were performed at 289.15 K (16 °C) and 303.15 K (30 °C). Root-mean-square deviation (RMSD), root-mean-square fluctuation (RMSF), radius of gyration (Rg), and solvent-accessible surface area (SASA) were computed using GROMACS built-in tools, and the resulting curves were plotted using Qtgrace (v0.2.6, open-source software). Two-dimensional and three-dimensional free-energy landscapes were generated using Python (v3.9, Python Software Foundation).

### 2.13. Statistical Analysis

All data are presented as mean ± standard deviation (SD). Data normality was assessed using the Shapiro–Wilk test, and homogeneity of variance was evaluated using Levene’s test. Student’s *t*-test and one-way ANOVA followed by Tukey’s post hoc test were applied when the assumptions of normality and homogeneity of variance were satisfied. Non-parametric tests were used when these assumptions were not met. Statistical analyses were performed using SPSS version 22.0. Differences were considered statistically significant at *p* < 0.05.

## 3. Results

### 3.1. Heat Stress Induces Oxidative Damage and Time-Dependent Apoptosis in C. gigas Haemocytes

**Figure 1:**
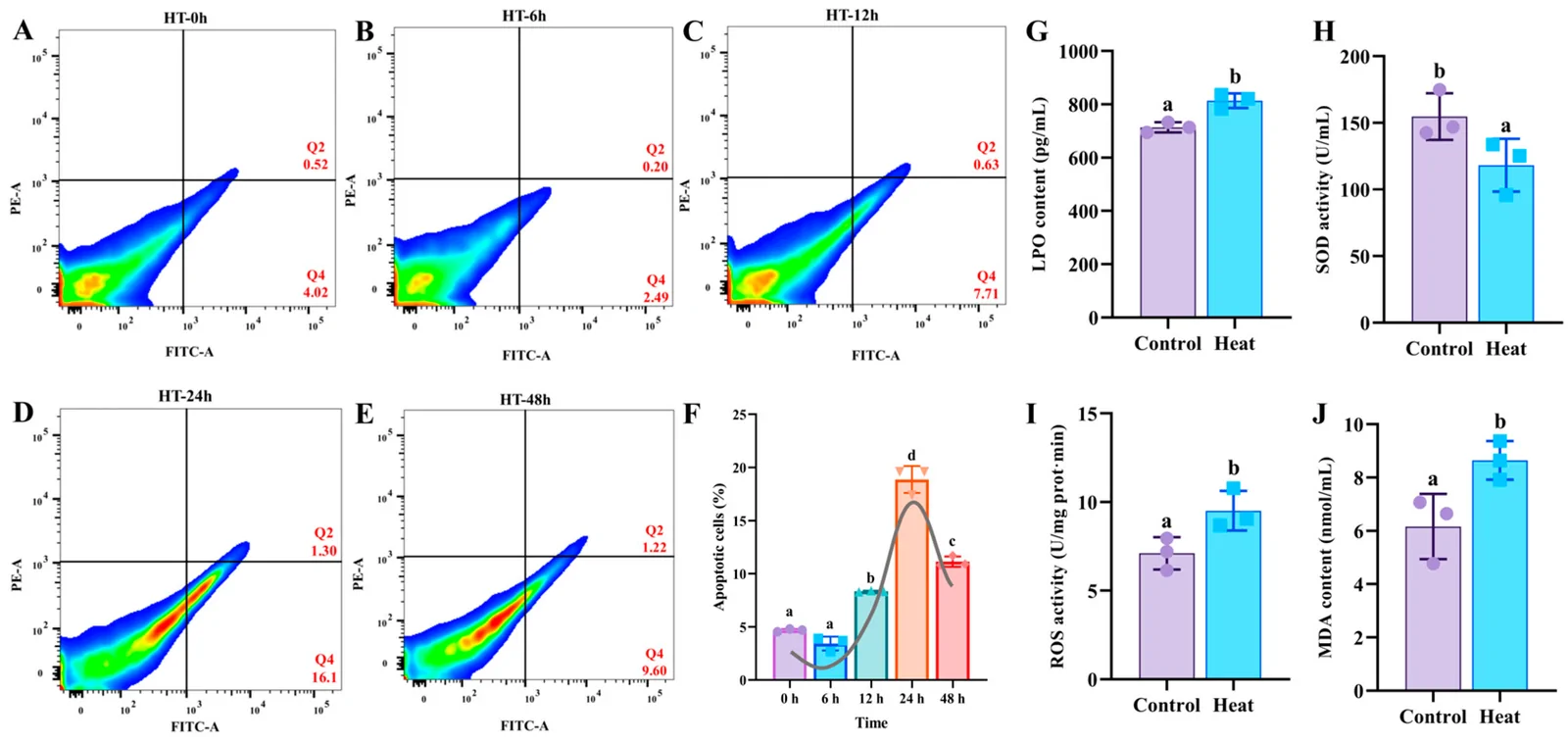
Heat stress induces apoptosis and oxidative damage in *C. gigas* haemocytes. (**A**–**E**) Annexin V-FITC/PI flow cytometry of haemocytes after 30 °C heat stress for 0, 6, 12, 24, and 48 h; Q2 and Q4 indicate late and early apoptotic cells. In the flow cytometry plots, red indicates higher cell density and blue indicates lower cell density. (**F**) Total apoptosis rate (Q2 + Q4) over time. (**G**–**J**) LPO content (**G**), SOD activity (**H**), ROS activity (**I**), and MDA content (**J**) in control (16 °C) versus heat-stressed (30 °C, 24 h) haemocytes. Mean ± SD (n = 3); different letters indicate *p* < 0.05.

The exposure at 30 °C triggered a coordinated pro-oxidant shift in *C. gigas* haemocytes. Relative to the 16 °C control, 24 h of 30 °C exposure significantly increased intracellular LPO, suppressed SOD activity, and increased ROS and MDA (*p* < 0.05; Figure 1G–J). This points to oxidative imbalance as the core cellular phenotype of heat injury, marked by ROS accumulation, lipid peroxidation, and antioxidant-enzyme suppression. The oxidative shift was accompanied by progressive apoptosis. Flow cytometry showed apoptosis remaining low at 0 and 6 h (4.54% and 2.69%; *p* > 0.05). It rose at 12 h (8.34%; *p* < 0.05), peaked at 24 h (17.40%; *p* < 0.01), and declined at 48 h (10.82%) (Figure 1A–F). Maximal apoptosis coincided with maximal oxidative injury at 24 h. This time point was therefore defined as the critical pathological window and selected for all subsequent transcriptomic and functional analyses.

### 3.2. Transcriptomic Profiling Identifies PDIA6 as the Central Hub Linking ER Stress, Apoptosis, and Oxidative Stress

**Figure 2:**
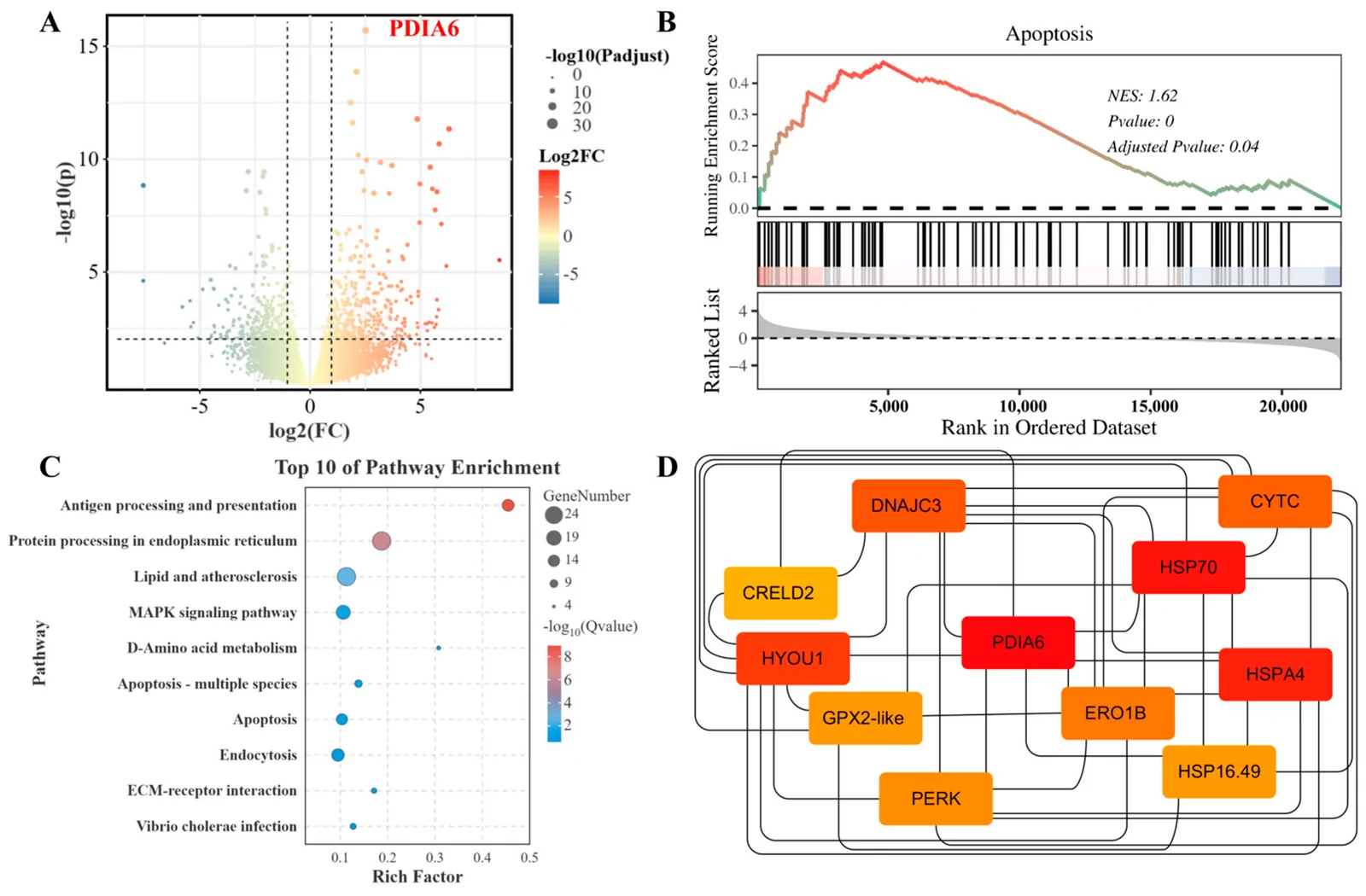
Transcriptomic identification of *Cg*PDIA6 as a hub regulator of heat-induced apoptosis and oxidative stress. (**A**) Volcano plot of DEGs between heat-stressed (30 °C, 24 h) and control haemocytes (|log2FC| ≥ 1, FDR < 0.05); *CgPDIA6* labeled in red. (**B**) GSEA enrichment plot for the apoptosis pathway (NES = 1.62, adjusted *p* = 0.04). The black dashed line indicates the baseline of running enrichment score equal to zero. (**C**) KEGG enrichment of DEGs (top 10 pathways). (**D**) PPI network identifying *Cg*PDIA6 as a central hub across the ER stress, apoptosis, and oxidative stress subnetworks.

To identify the upstream regulator coupling heat stress to this oxidative phenotype, haemocyte transcriptomes from heat-stressed (30 °C, 24 h) and control (16 °C) oysters were compared. Among the differentially expressed genes (DEGs) resolved by volcano analysis, *CgPDIA6* was one of the most strongly upregulated transcripts (Figure 2A). Consistent with a coordinated death program, GSEA ranked the apoptosis pathway highest of all gene sets (NES = 1.62, adjusted *p* = 0.04; Figure 2B), and KEGG enrichment placed protein processing in the endoplasmic reticulum among the top pathways (Figure 2C), directly linking ER proteostasis to the heat response. To locate the regulatory core of these converging programs, a PPI network was built from the union of the top 100 high-confidence DEGs, the leading-edge genes of the top 20 GSEA pathways, and all genes of the top 10 KEGG pathways (Figure 2D). In this network *Cg*PDIA6 emerged as the hub with the highest node degree, interacting directly with ER-stress and redox effectors including ERO1B, PERK, HSP70, HSPA4, DNAJC3, GPX2-like, and HYOU1. *Cg*PDIA6 was therefore identified as the central node integrating ER stress, apoptosis, and oxidative stress in heat-stressed haemocytes and was prioritized for functional dissection.

### 3.3. Molecular Characterization, Phylogeny, and Tissue/Developmental Transcript Expression of CgPDIA6

**Figure 3:**
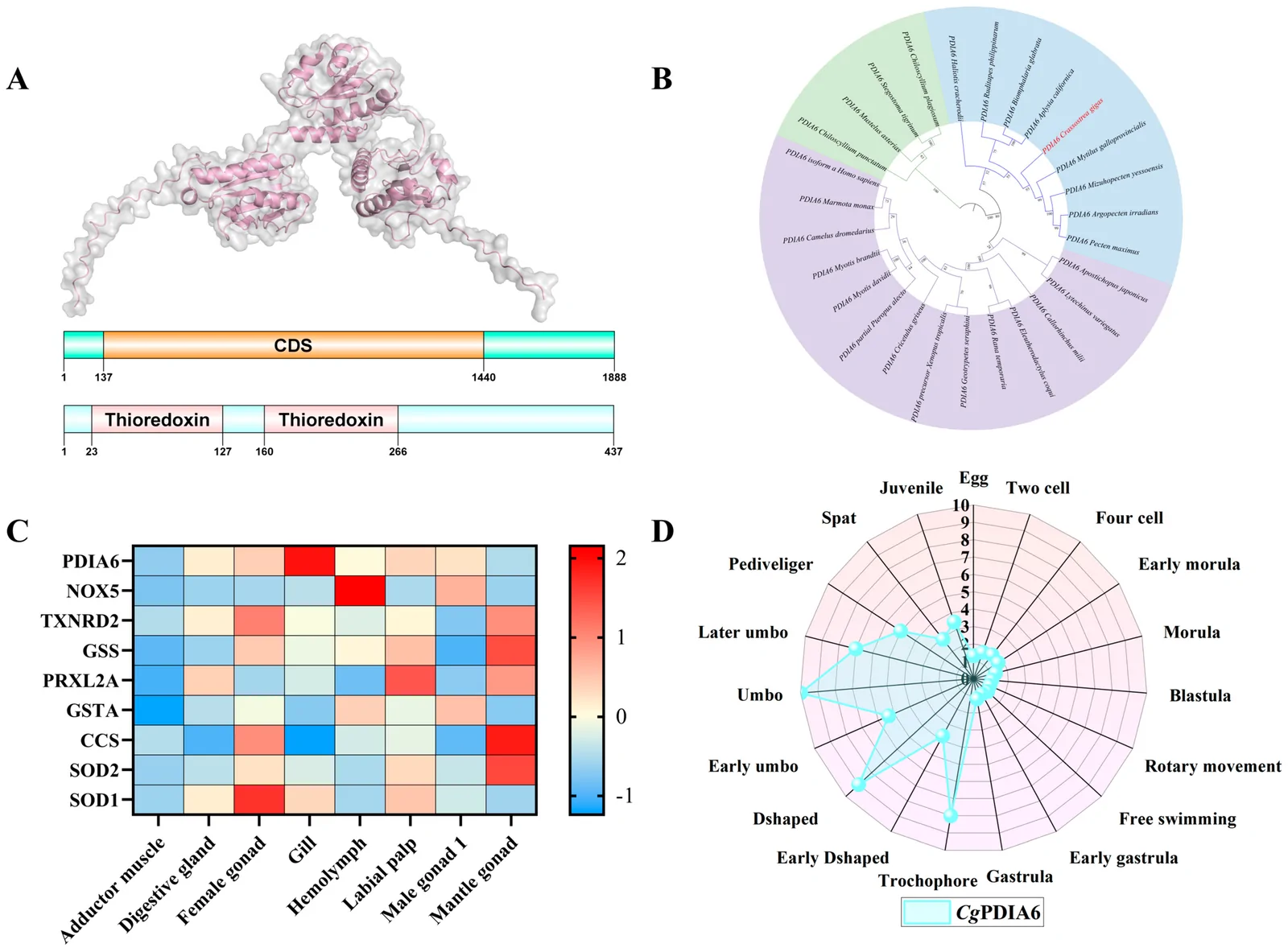
Molecular characterization, phylogeny, and expression profiling of *CgPDIA6*. (**A**) Schematic of the *CgPDIA6* mRNA architecture and the predicted three-dimensional structure and conserved domain organization of the *Cg*PDIA6 protein, which contains two tandem thioredoxin (TRX) domains. (**B**) Neighbor-joining phylogenetic tree of PDIA6 protein sequences from 27 representative species; *C. gigas* is highlighted in red. (**C**) Heatmap (Z-score) of *CgPDIA6* transcript abundance and oxidative stress-related genes across eight adult tissues. (**D**) Radar chart of *CgPDIA6* expression (FPKM) across 20 developmental stages.

The *Cg*PDIA6 protein contained two tandem thioredoxin (TRX) domains (Figure 3A), reflecting the typical architecture of the PDI family. In the phylogenetic tree, *Cg*PDIA6 clustered with other bivalves (Figure 3B), indicating that PDIA6 is evolutionarily conserved. Tissue profiling across eight adult tissues showed that *CgPDIA6* transcript abundance was highest in haemocytes (FPKM = 547), the circulating immune effectors most exposed to heat-induced oxidative challenge. In these cells, *CgPDIA6* showed coordinated transcript patterns with antioxidant-related genes including *CgSOD1*, *CgSOD2, CgGSTA*, and *CgTXNRD2* (Figure 3C), suggesting a potential association with antioxidant-related responses. Across development, *CgPDIA6* was low in early embryos but rose markedly from the D-shaped larval stage onward, remaining elevated through the spat and juvenile stages (Figure 3D). This pattern is consistent with an increasing demand for ER redox homeostasis as environmental exposure increases. These profiles support a dedicated antioxidant-protective role for *CgPDIA6* in haemocytes.

### 3.4. In Silico CgPDIA6 Knockout Exposes a Haemocyte Antioxidant Network

**Figure 4:**
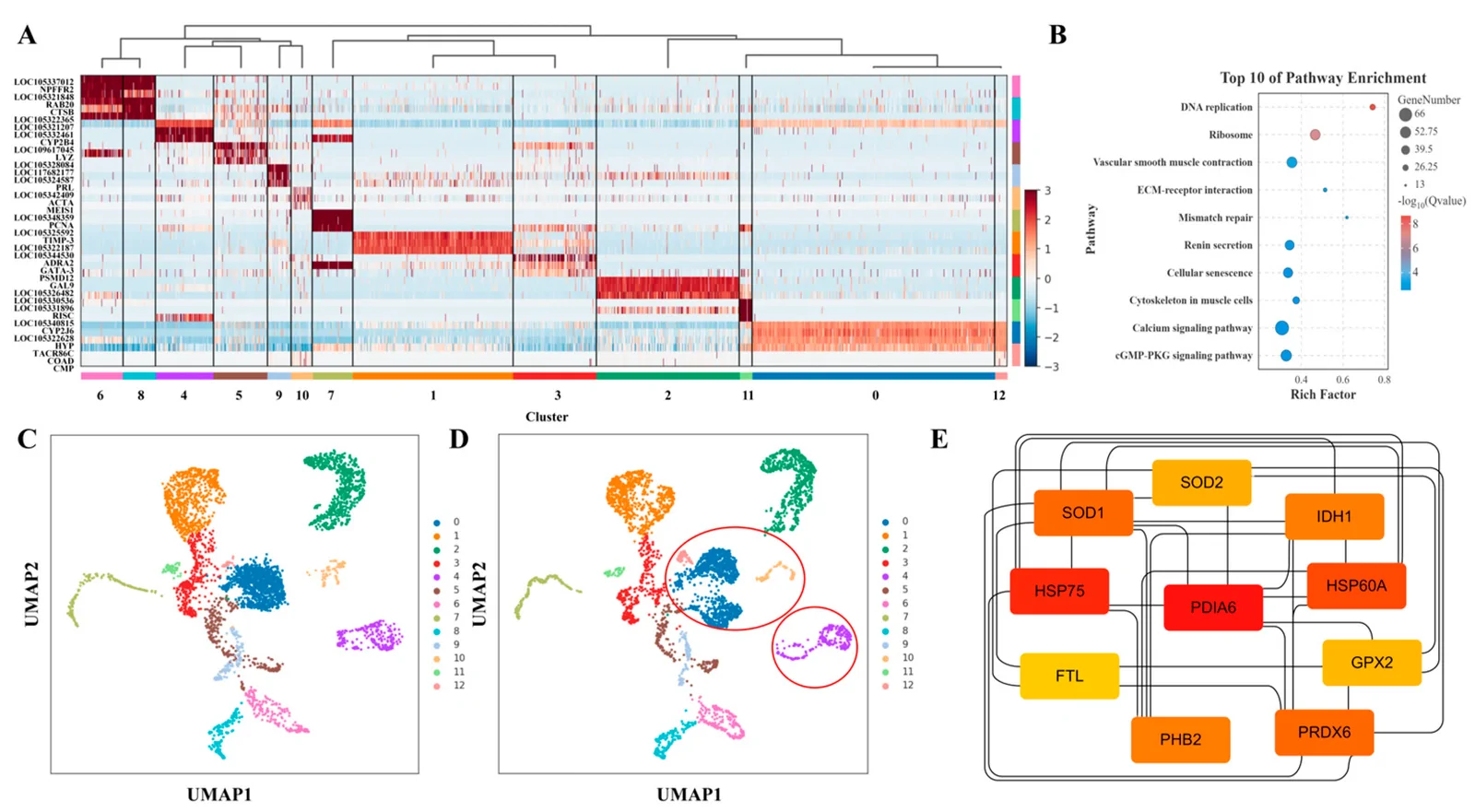
Single-cell analysis of in silico *Cg*PDIA6 knockout in *C. gigas* haemocytes. (**A**) Marker gene heatmap across 13 haemocyte clusters (0–12). The bottom color bars represent distinct haemocyte clusters, and the color gradient on the right indicates normalized gene expression levels. (**B**) KEGG enrichment of the top 5000 highly variable genes under knockout (top 10 pathways). (**C**,**D**) UMAP of haemocyte clusters in wild-type (**C**) and *Cg*PDIA6 knockout (**D**); red circles mark significantly shifted clusters. (**E**) PPI network of the shifted clusters, linking *Cg*PDIA6 to antioxidant (*Cg*SOD1, *Cg*SOD2, *Cg*GPX2, *Cg*PRDX6, *Cg*FTL) and stress-response (*Cg*HSP75, *Cg*HSP60A, *Cg*IDH1, *Cg*PHB2) effectors.

To probe the consequences of *Cg*PDIA6 perturbation, an in silico knockout was performed on single-cell haemocyte transcriptomes by computationally removing all PDIA6-associated interaction edges. The most informative result was the downstream effector network. A secondary PPI network built from the most strongly affected clusters placed *Cg*PDIA6 at a central node directly connected to the antioxidant effectors *Cg*SOD1, *Cg*SOD2, *Cg*GPX2, *Cg*FTL, and *Cg*PRDX6 and to the iron- and mitochondria-linked stress proteins *Cg*PHB2, *Cg*IDH1, *Cg*HSP75, and *Cg*HSP60A (Figure 4E). This analysis suggested a potential mechanism associated with *Cg*PDIA6-mediated redox regulation. Following computational disruption of the *Cg*PDIA6-associated network, the predicted haemocyte states showed a shift from homeostatic toward stress-activated profiles. The predicted stress-activated effector population (Cluster 12) increased from 24 to 83 cells, while the resting and cytoskeletal/developmental populations (Clusters 0 and 5) decreased (Figure 4A,C,D; Table 1). KEGG analysis of the knockout-responsive genes revealed enrichment of cellular senescence and related stress pathways (Figure 4B). Together, these analyses predict that *Cg*PDIA6 perturbation may influence a multi-component antioxidant network and may contribute to stress-associated haemocyte state transitions.

### 3.5. RNAi Knockdown of CgPDIA6 Amplifies Heat-Induced Oxidative Damage

**Figure 5:**
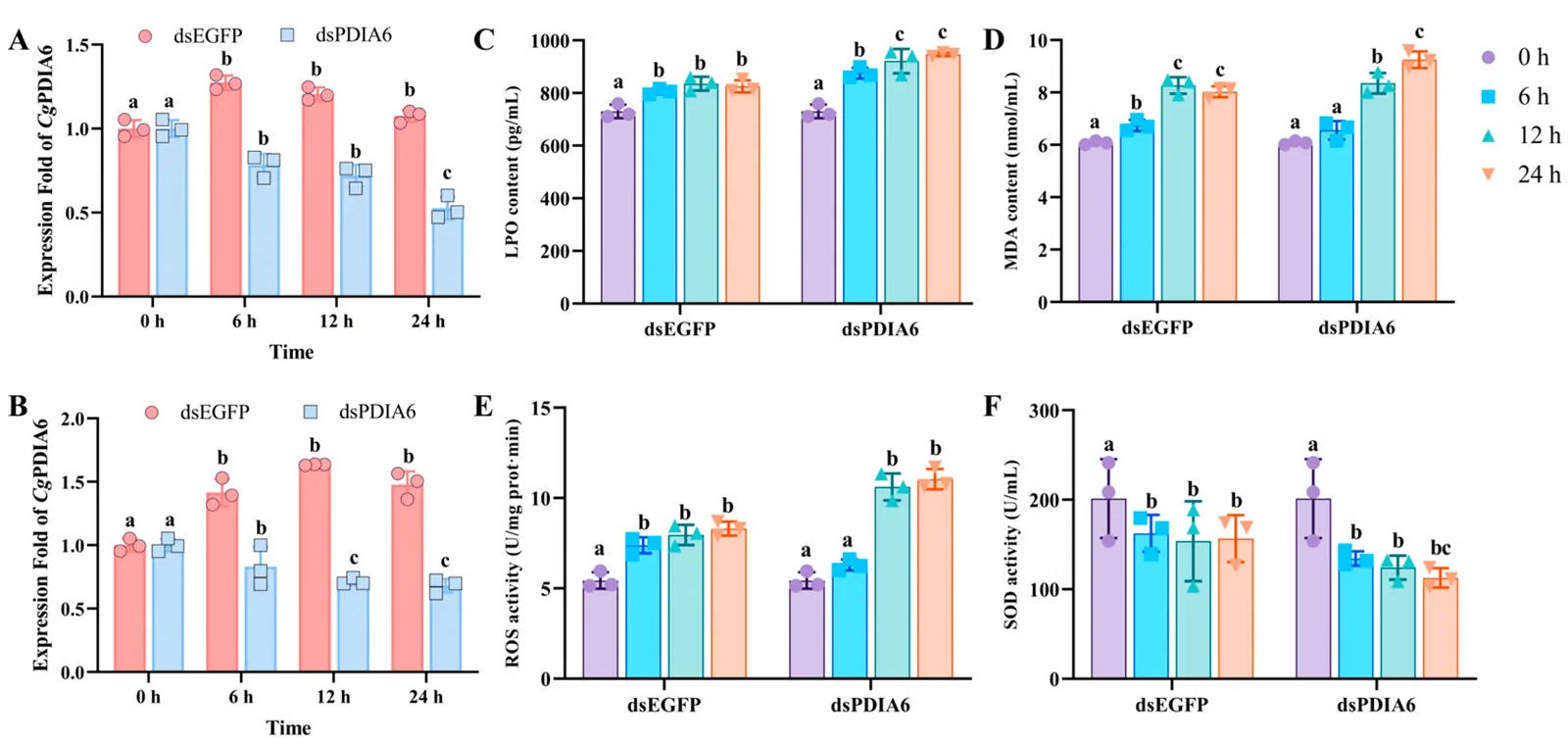
The inhibition of *CgPDIA6* expression by RNAi aggravates heat-induced oxidative damage. (**A**,**B**) *CgPDIA6* knockdown efficiency (dsPDIA6 vs. dsEGFP) at 16 °C (**A**) and 30 °C (**B**). (**C**–**F**) Oxidative stress markers in the dsEGFP and dsPDIA6 groups at 0, 6, 12, and 24 h under 30 °C stress: (**C**) LPO content, (**D**) MDA content, (**E**) ROS activity, and (**F**) SOD activity. Mean ± SD (n = 3); different letters indicate *p* < 0.05.

To test this prediction experimentally, *CgPDIA6* was silenced by RNAi. qRT-PCR confirmed effective knockdown. Under 16 °C conditions, dsPDIA6 reduced *CgPDIA6* mRNA expression by 47.7% at 24 h compared with the dsEGFP control (Figure 5A). Under 30 °C heat stress, *CgPDIA6* expression was reduced by more than 60% at 12 and 24 h after dsPDIA6 treatment (Figure 5B). *CgPDIA6* knockdown aggravated heat-induced oxidative damage. Compared with dsEGFP controls, dsPDIA6 haemocytes showed significantly increased ROS levels at all examined time points (Figure 5E). LPO and MDA contents were significantly elevated after knockdown, with the largest differences observed at 24 h (Figure 5C,D). SOD activity showed a decreasing trend after *CgPDIA6* knockdown, with the lowest level observed at 24 h (Figure 5F). To determine whether *CgPDIA6* affects oxidative status under non-stress conditions, related parameters were examined at 16 °C. No significant differences in LPO, MDA, ROS levels, or SOD activity were observed between dsPDIA6 and dsEGFP groups (Figure S1). These results indicate that *CgPDIA6* knockdown mainly affects oxidative responses under heat stress.

### 3.6. CgPDIA6 Knockdown Promotes Apoptosis and Alters Antioxidant Responses

**Figure 6:**
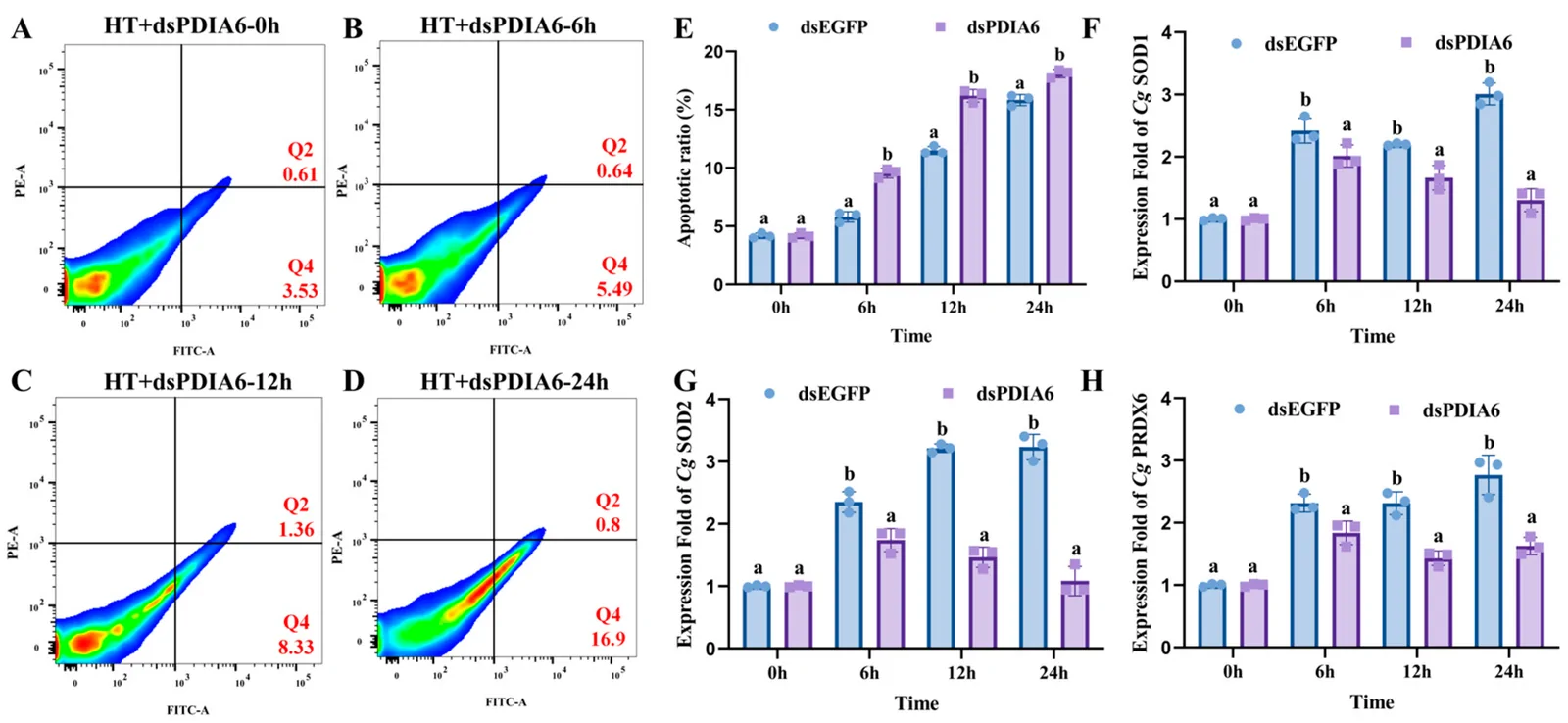
*CgPDIA6* knockdown enhances haemocyte apoptosis and alters antioxidant gene expression under heat stress. (**A**–**D**) Representative Annexin V-FITC/PI flow cytometry analysis of haemocyte apoptosis in dsPDIA6-injected oysters under 30 °C heat stress at 0, 6, 12, and 24 h. Q2 and Q4 indicate late and early apoptotic cells, respectively. In the flow cytometry plots, red indicates higher cell density and blue indicates lower cell density. (**E**) Quantification of total apoptotic rates (Q2 + Q4) in dsEGFP and dsPDIA6 groups during 30 °C exposure. (**F**–**H**) Relative expression levels of antioxidant-related genes after *CgPDIA6* knockdown under heat stress: (**F**) *CgSOD1*, (**G**) *CgSOD2*, and (**H**) *CgPRDX6*. Data are presented as mean ± SD (n = 3). Different letters indicate significant differences (*p* < 0.05).

To further evaluate the effects of *CgPDIA6* knockdown under heat stress, haemocyte apoptosis and antioxidant-related gene expression were analyzed after RNAi treatment. Compared with dsEGFP controls, dsPDIA6 haemocytes showed significantly increased apoptosis rates during 30 °C exposure (Figure 6A–E). The difference became more evident with prolonged heat stress, and the apoptosis rate reached the highest level at 24 h, with 17.7% in the dsPDIA6 group. In addition, *CgPDIA6* knockdown significantly affected the expression of antioxidant-related genes. The expression levels of *CgSOD1*, *CgSOD2*, and *CgPRDX6* were reduced in dsPDIA6 haemocytes compared with dsEGFP controls (Figure 6F–H). Among these genes, *CgSOD2* exhibited the strongest response, showing a greater decrease than *CgSOD1* during heat stress. These results reveal that *CgPDIA6* knockdown is accompanied by increased haemocyte apoptosis and altered expression patterns of antioxidant-related genes.

### 3.7. Molecular Dynamics Simulation Reveals Heat-Induced Destabilization of CgPDIA6

**Figure 7:**
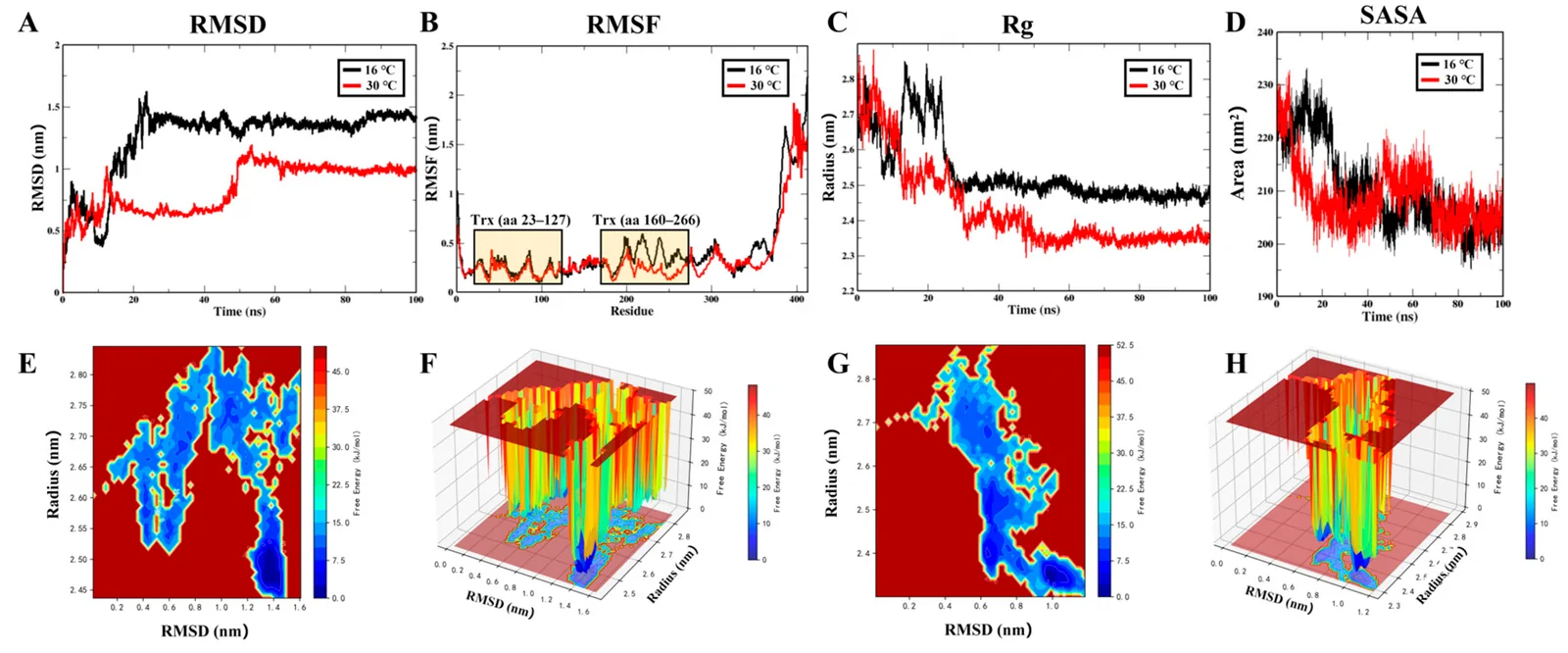
Molecular dynamics simulation of *Cg*PDIA6 at 16 °C and 30 °C. (**A**) Backbone RMSD, (**B**) per-residue RMSF, (**C**) radius of gyration (Rg), and (**D**) solvent-accessible surface area (SASA) over the 100-ns trajectories (16 °C, black; 30 °C, red). (**E**,**F**) Two- and three-dimensional free-energy landscapes (FEL) at 16 °C. (**G**,**H**) Corresponding free-energy landscapes at 30 °C. Axes represent RMSD and radius of gyration.

To assess heat effects on *Cg*PDIA6, 100-ns MD simulations were performed at 16 °C and 30 °C. Both trajectories reached a stable RMSD plateau (Figure 7A), and the radius of gyration converged to similar values (Figure 7C), indicating that the overall fold was maintained. However, the 30 °C system showed clearly higher per-residue fluctuations than the 16 °C control, including within the two thioredoxin catalytic domains (aa 23–127 and 160–266) (Figure 7B), together with a larger solvent-accessible surface area (Figure 7D). Consistently, the free-energy landscape broadened from a single narrow basin at 16 °C (Figure 7E,F) into a wider, multi-welled region at 30 °C (Figure 7G,H). These results indicate that heat increases the local flexibility and conformational instability of *Cg*PDIA6, particularly around its catalytic domains, suggesting a destabilizing effect on protein function.

## 4. Discussion

The molecular regulators coupling heat stress to redox dysregulation in bivalve immune cells remain poorly defined. This study identifies the ER oxidoreductase *Cg*PDIA6 as a redox-protective hub linking heat stress to oxidative damage in *C. gigas* haemocytes. The following sections discuss how *Cg*PDIA6 coordinates the ER stress–oxidative stress–apoptosis axis, sustains a haemocyte antioxidant network, and is itself destabilized by heat at its catalytic domains.

The biphasic apoptotic response of *C. gigas* haemocytes reflects the limited capacity of an adaptive stress programme. Sublethal heat first activates survival pathways that restrain cell death [32]. As stress persists, these defences are overwhelmed and apoptosis rises, peaking at 24 h before a partial recovery at 48 h that reflects clearance of damaged cells rather than true resolution [32]. The heat-induced upregulation of *CgPDIA6* is one component of this adaptive buffer, which delays but cannot indefinitely prevent death under sustained heat load. Comparable biphasic kinetics in *Sinonovacula constricta*, where heat activates p38-MAPK and caspase-3 before a secondary adaptive response [16], indicate that this finite-buffer dynamic is conserved among bivalves. The timing of the response is itself informative. Peak apoptosis coincides precisely with maximal ROS, MDA, and LPO and minimal SOD activity, a convergence that identifies oxidative stress as the driver of heat apoptosis rather than its by-product. This establishes 24 h as the critical window for dissecting the upstream control of haemocyte redox failure.

Heat stress accelerates ER protein misfolding, and the resulting overload of the ERO1–PDI folding cycle makes the ER itself a source of ROS [33]. This positions PDIA6 at the interface between protein folding and redox control. A position at this interface alone, however, does not explain why PDIA6 rather than another folding enzyme emerged as the network hub. The distinction lies in a regulatory function absent from the purely catalytic members of the PDI family. PDIA6 limits the duration of the UPR by binding the oxidized Cys148 of IRE1α and attenuating PERK signalling, which restrains apoptotic commitment, whereas its loss sustains IRE1α autophosphorylation and ROS accumulation [10,11]. PDIA6 is therefore not merely a folding catalyst but a checkpoint that couples the severity of ER proteostatic stress to the decision between survival and apoptosis. The present network supports this role, as PDIA6 interacted directly with ERO1B, the principal hydrogen peroxide-generating oxidase of the ER lumen [34], and co-clustered with the ER chaperone module including HSP70, HYOU1, and DNAJC3. Its conserved heat induction across *S. constricta*, *A. japonicus*, and *A. cerana* indicates that this checkpoint function is not specific to the oyster but represents an evolutionarily general strategy for coupling proteostasis to redox defence under heat stress [35,36,37].

The tissue and developmental distribution of *CgPDIA6* indicates that it functions as a dedicated antioxidant factor rather than a constitutive housekeeping enzyme. *CgPDIA6* was enriched in haemocytes, the immune cells most exposed to heat-induced oxidative challenge, rather than in the gill or digestive gland that handles systemic detoxification [17]. This pattern points to a cell-autonomous redox role tied to immune activity rather than to bulk metabolic clearance. *CgPDIA6* showed coordinated transcript patterns with *SOD1*, *SOD2*, *GSTA*, and *TXNRD2* in haemocytes. These patterns suggest that *CgPDIA6* may be associated with antioxidant-related responses. Its expression also rose sharply from the D-shaped larval stage onward, the point at which active feeding and shell formation impose steep metabolic and protein-folding loads and a robust ER antioxidant capacity first becomes essential [38]. Across both tissue and developmental axes, therefore, *CgPDIA6* is expressed specifically where oxidative folding demand is highest. This distribution is difficult to reconcile with a housekeeping role and instead marks it as a stress-responsive guardian of haemocyte redox homeostasis.

Antioxidant defence proceeds as an ordered enzymatic cascade rather than through any single enzyme [39]. Locating *Cg*PDIA6 within this cascade therefore clarifies how its loss translates into oxidative failure. In the in silico network, *Cg*PDIA6 connected directly to the superoxide dismutases *Cg*SOD1 and *Cg*SOD2 and to the peroxiredoxin *Cg*PRDX6, the enzymes that carry out two consecutive steps of this cascade: the dismutases convert superoxide into hydrogen peroxide, which *Cg*PRDX6 then reduces to water [40]. Because *Cg*PDIA6 was positioned upstream of these antioxidant enzymes in the predicted network, *Cg*PDIA6 perturbation may influence this antioxidant relay. The RNAi time course supports this interpretation. The reduced transcript levels of *CgSOD1*, *CgSOD2*, and *CgPRDX6* after *CgPDIA6* knockdown further support the association between *CgPDIA6* and antioxidant-related responses. Rather than a single marker rising in isolation, all four oxidative indicators deteriorated in a coordinated, graded manner, with ROS rising first, lipid peroxidation peaking at 24 h, and SOD activity declining progressively from 12 h. This coordinated response is consistent with a broad disruption of antioxidant capacity rather than the alteration of a single oxidative marker. It indicates that *Cg*PDIA6 does not abolish oxidative injury but delays it by holding the cascade intact. Removing *Cg*PDIA6 therefore unmasks the full trajectory of damage. The current evidence is derived from systemic RNAi and transcript-level analysis. Further studies using targeted cellular approaches and protein-level validation are needed to clarify the precise role of *CgPDIA6* in haemocyte regulation.

Beyond raising the cellular demand for *Cg*PDIA6, heat may also affect directly on the enzyme itself. In the 100-ns simulations, the global fold of *CgPDIA6* was preserved at 30 °C, as shown by the stable RMSD plateau and converged radius of gyration. Despite this preserved fold, its two thioredoxin domains became markedly more flexible. They showed higher per-residue fluctuations, greater solvent exposure, and a broadened free-energy landscape relative to 16 °C. These domains harbour the *Cg*HC catalytic motifs, whose activity depends on a precisely constrained active-site geometry [41]. The increased local flexibility is therefore expected to impair catalytic efficiency, even while the protein remains globally folded. Heat thus does not simply raise the demand on *CgPDIA6*. It also erodes the catalytic competence of each molecule. This dual action reconciles the heat-induced upregulation of *CgPDIA6* with the oxidative injury that still develops. The upregulation is a compensatory response. But heat destabilization undercuts the very enzyme being mobilized, so this compensation remains incomplete, and the protective buffer is eventually outpaced. The simulation complements the RNAi data. Knockdown showed that lowering *Cg*PDIA6 abundance aggravates oxidative damage. The MD analysis indicates that heat itself degrades *Cg*PDIA6 function. Both therefore converge on a deficit of functional enzyme. While this predicted loss of activity was inferred from simulation, it awaits direct enzymatic validation. By compromising *CgPDIA6* at its catalytic core, heat weakens a key antioxidant safeguard and intensifies the oxidative damage that drives haemocyte apoptosis.

## 5. Conclusions

This study identifies the ER oxidoreductase *Cg*PDIA6 as a key redox-protective regulator linking heat stress to oxidative damage in Pacific oyster haemocytes. Heat stress drove a coordinated oxidative shift that culminated in apoptosis, and *Cg*PDIA6 emerged as the central hub coupling ER stress, apoptosis, and oxidative stress. Functional analyses showed that *Cg*PDIA6 sustains haemocyte antioxidant defence and that its loss aggravates heat-induced oxidative injury. *Cg*PDIA6 knockdown reduced the expression of antioxidant-related genes, including *CgSOD1*, *CgSOD2*, and *CgPRDX6*, further supporting its role in maintaining redox homeostasis. Heat further destabilizes the catalytic core of *Cg*PDIA6, simultaneously increasing the demand on this enzyme and undermining the protection it provides. *Cg*PDIA6 therefore coordinates the ER stress–oxidative stress–apoptosis axis as a checkpoint whose own heat vulnerability sets a limit on haemocyte resilience. These findings identify *Cg*PDIA6 as a mechanistically grounded redox marker and a candidate target for improving heat resilience in oyster aquaculture.

## Figures and Tables

**Table 1:** *CgPDIA6* knockout alters haemocyte subpopulation composition.

Cluster	WT	KO	Change	Change_pct	Marker Gene	Description
0	1070	1030	−40	−3.74%	*LOC105337012* *NPFFR2* *LOC105321848*	Neuroendocrine-like resting haemocyte
1	676	711	35	5.18%	*RAB20* *CTSB* *LOC105322365*	Lysosomal/phagocytic haemocyte
2	616	624	8	1.30%	*LOC105321207* *LOC105332461* *CYP2B4*	Cytoprotective/metabolic haemocyte
3	353	366	13	3.68%	*LOC109617045* *LYZ* *LOC105328084*	Antimicrobial/lysozyme-producing haemocyte
4	252	249	−3	−1.19%	*LOC117682177* *LOC105324587* *PRL*	Apoptosis-primed/prolactin-related haemocyte
5	257	210	−47	−18.29%	*LOC105342409* *ACTA* *MEIS1*	Cytoskeletal/developmental haemocyte
6	181	184	3	1.66%	*LOC105348359* *PCNA* *LOC105325592*	Proliferating/hematopoietic progenitor haemocyte
7	177	172	−5	−2.82%	*TIMP-3* *LOC105322187* *LOC105344530*	ECM-remodelling/tissue repair haemocyte
8	141	142	1	0.71%	*ADRA2* *GATA-3* *PSMD12*	Stress-signalling/neuroendocrine haemocyte
9	116	87	−29	−25.00%	*GAL9* *LOC105326482* *LOC105330536*	Immunomodulatory/galectin-expressing haemocyte
10	87	99	12	13.79%	*LOC105331896* *RISC* *LOC105340815*	RNA interference/regulatory haemocyte
11	58	51	−7	−12.07%	*CYP2J6* *LOC105322628* *HYP*	ROS-metabolizing/cytochrome P450 haemocyte
12	24	83	59	245.83%	*TACR86C* *COAD* *CMP*	Stress-activated effector haemocyte

## Data Availability

The raw data supporting the conclusions of this article will be made available by the authors on reasonable request.

## Supplementary Materials

The following supporting information can be downloaded at https://www.mdpi.com/article/10.3390/antiox15080963/s1: Table S1: Primer sequences used in this study; Figure S1: *Cg*PDIA6 knockout alters haemocyte subpopulation composition.

## Abbreviations

The following abbreviations are used in this manuscript:

ROS	Reactive oxygen species
MDA	Malondialdehyde
LPO	Lipid hydroperoxide
SOD	Superoxide dismutase
IPTG	Isopropyl β-D-1-thiogalactopyranoside
PDIA6	disulfide isomerase A6 Protein
*C. gigas*	*Crassostrea gigas*
