# Supplementary material for: Protein Disulfide Isomerase A6 (PDIA6) Restrains Heat-Induced Oxidative Damage in Haemocytes of the Pacific Oyster (Crassostrea gigas)
Source: Antioxidants (Basel). 2026 Aug 1;15(8):963. doi: 10.3390/antiox15080963 (PMC13509791; doi:10.3390/antiox15080963)
Supplement: Supplementary file 1 [file antioxidants-15-00963-s001.zip › Supplementary Figure S1.pdf]

Supplementary Figure

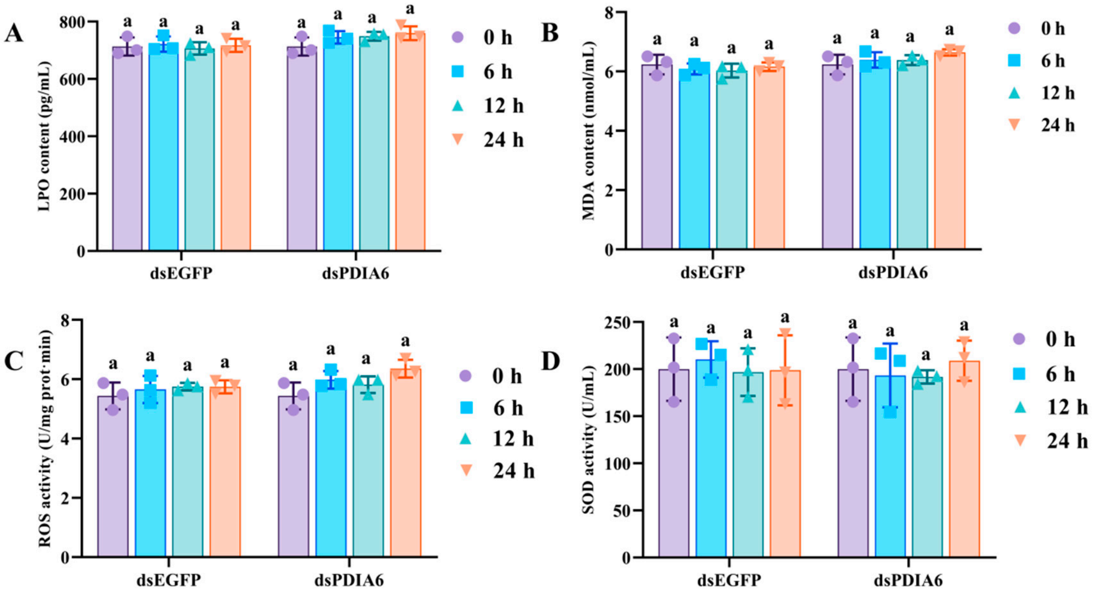

Figure S1. Effects of *Cg*PDIA6 knockdown on oxidative stress-related parameters under 16 °C conditions.(A－D) Oxidative stress-related parameters in haemocytes after *Cg*PDIA6 knockdown at 16 °C: (A) LPO content, (B) MDA content, (C) ROS activity, and (D) SOD activity. The dsEGFP group was used as the control. Data are presented as mean ± SD (n = 3). Different letters indicate significant differences ($p < 0.05$).
