# Supplementary material for: Protein Disulfide Isomerase A6 (PDIA6) Restrains Heat-Induced Oxidative Damage in Haemocytes of the Pacific Oyster (Crassostrea gigas)
Source: Antioxidants (Basel). 2026 Aug 1;15(8):963. doi: 10.3390/antiox15080963 (PMC13509791; doi:10.3390/antiox15080963)
Supplement: Supplementary file 1 [file antioxidants-15-00963-s001.zip › Supplementary Table S1.pdf]

Supplementary Table S1. Primer sequences used in this study.

Primers	Sequence (5′-3′)
ds-PDIA6-F	AAGCTTTCTCTGGTTCAGGACAGGCT
ds-PDIA6-R	GCTAGCATATCCTCGGACCCCGTATC
RT-SOD1-F	GGCGAAGACGGTGTCGCCAA
RT-SOD1-R	AAGCCAATCGTCCGCCAGCA
RT-SOD2-F	GTGCCCTGGGAGCGATGGG
RT-SOD2-R	GCCTCTGCCAGTTTCTCCTCAGC
RT-PRDX6-F	CCCAGCAACCACTGGCCGAA
RT-PRDX6-R	GAGGGTACGGGCTGGACGGT
RT-PDIA6-F	TGAGGCAGCACTCTGCCAGT
RT-PDIA6-R	ACTGTCCAGCACCAGCCGCT
